# The efficacy and safety of evogliptin for type 2 diabetes mellitus: A systematic review and meta-analysis

**DOI:** 10.3389/fendo.2022.962385

**Published:** 2022-08-19

**Authors:** Qizhi Tang, Weiyu Pan, Liangyue Peng

**Affiliations:** Department of Endocrinology, Guangdong Hospital of Integrated Traditional Chinese and Western Medicine, Foshan, China

**Keywords:** diabetes, glucose intolerance, dipeptidyl peptidase-4 inhibitor, adverse events, metabolic

## Abstract

**Introduction:**

The dipeptidyl peptidase-4 inhibitor (DPP-4i) drugs, such as evogliptin, as the second-line drugs for type 2 diabetes mellitus (T2DM) treatment have been reported to facilitate insulin secretion by reducing glucagon and inhibiting glucagon like peptides. With a vague consensus, the advantageous and non-inferior effects of evogliptin relative to other DPP-4i drugs were recently demonstrated on hemoglobin A1c (HbA1c) levels and overall adverse events in T2DM patients. Thus, the aim was to evaluate the overall influence of evogliptin on HbA1c levels and the adverse events in T2DM patients compared to sitagliptin and linagliptin.

**Methods:**

Complying with PRISMA guidelines, we conducted a systematic literature search in databases and a meta-analysis. Data about HbA1c levels and the adverse events of T2DM patients were collected and analyzed.

**Results:**

From 1,397 studies, we found five matched studies involving 845 subjects (mean age: 54.7 ± 3 years). The meta-analysis revealed that evogliptin was non-inferior to sitagliptin/linagliptin with a mean difference of 0.062 (95% CI: -0.092 to 0.215. I^2^: 0%. *P* = 0.431) regarding the HbA1c level reduction, and the risk ratio was -0.006 (95% CI: -0.272 to 0.260. I^2^: 1.7%. *P* = 0.966) regarding the adverse effects, indicating no significant difference between evogliptin and linagliptin or sitagliptin in affecting the HbA1c level and adverse effects.

**Conclusion:**

The study provides preliminary evidence regarding the similarity in the efficacy of evogliptin compared to other DPP-4i drugs, including sitagliptin and linagliptin, for managing HbA1c levels and adverse events.

## Introduction

As a metabolic disorder commonly observed across the world, Type 2 diabetes mellitus (T2DM) is characterized by an increased level of fasting glucose beyond 7.0 mmol/L or ≥6.5% levels of hemoglobin A1c (HbA1c) (1). Epidemiological research has widely documented a high prevalence rate of 12.8% of DM among adults aged 20–79 years old (2), and as per recent disease statistics, diabetes contributes to nearly 4.6 million deaths annually across the world (3). T2DM progresses relentlessly in terms of severity due to the insensitivity or inadequacy of natural insulin for metabolizing carbohydrates and fat (4). Here, persistent high blood glucose levels, predominantly due to the natural familial history of T2DM, are likely due to aggravation in β-cell defects, which eventually promote the disease severity (4). T2DM patients manifest numerous complications that significantly influence the prognosis of mortality and long-term life quality.

There are currently many conventional drugs for the treatment of T2DM, such as metformin, thiazolidinediones, sulfonylureas, dipeptidyl peptidase-4 inhibitor (DPP-4i), and sodium-glucose cotransporter-2 inhibitor (5). However, every medicine has its drawbacks and advantages. Some complications of T2DM patients, such as severe diabetic nephropathy, limit the application of some of the above drugs. Therefore, it is of great significance to search for more novel drugs and determine their application scope for diversifying the choices for T2DM patients. Among those drugs, DPP-4i, as an anti-hyperglycemic drug that can regulate the incretin hormones to enhance glucose tolerance and increase insulin levels by inhibiting the loss of β-cells mass, has been recommended as one of the second-line agents to treat T2DM. Evogliptin, a new DPP-4i drug marketed in 2015, has a superior influence in increasing the glucagon-like peptide-1 levels, which eventually results in reduced glucose levels within a durational period (6), and can bypass renal metabolization as compared to other DPP-4i drugs such as sitagliptin and linagliptin (7).

Due to the relatively short period of clinical application of evogliptin, only a few studies have reported its effect on HbA1C levels and adverse events. Furthermore, several individual randomized controlled studies are already evaluating the efficacy of evogliptin in managing T2DM outcomes in terms of HbA1c levels compared to other DPP-4i drugs, including sitagliptin and linagliptin. Here, some studies have reported a higher efficacy of evogliptin as compared to linagliptin (8) and sitagliptin (9), for reducing HbA1c levels and decreasing the number of overall adverse event outcomes, whereas the others have reported opposite results (10, 11). These differential results and a lack of consensus regarding the efficacy of evogliptin compared to other DPP-4i drugs, including sitagliptin and linagliptin for managing T2DM, have prevented diabetologists worldwide from effectively establishing optimal practice guidelines of DPP-4i drugs for managing T2DM. In addition, there is currently no systematic review or meta-analysis that evaluates the overall influence of evogliptin on HbA1c levels and the adverse events in T2DM patients compared to other DPP-4i drugs, including sitagliptin and linagliptin.

Since evogliptin differs from other DPP-4i medications such as sitagliptin and linagliptin in terms of its overall impact on HbA1c levels and the adverse events in T2DM patients, we aimed to integrate this information in the current study and evaluate the advantages or non-inferior effects of evogliptin on reducing the HbA1c level and adverse events. Our findings may enhance diabetologists’ clinical knowledge of T2DM care, while minimizing pertinent complications and providing support for one more choice for diabetic patients.

## Materials and methods

A systematic review and meta-analysis were performed complying with the PRISMA guideline (12).

### Data search strategy

The databases, including MEDLINE, CENTRAL, EMBASE, and Scopus, were searched from inception until October 2020 with the below-searched MeSH keywords “Type 2 diabetes”, “diabetes mellitus”, “Evogliptin”, “Sitagliptin”, “Linagliptin”, “HbA1c”, and “adverse events” in different combinations. Furthermore, we followed the inclusion criteria below to manually screen the included research’s bibliography parts to ascertain further relevant studies.

a) Studies examined HbA1c outcomes in T2DM patients receiving evogliptin, sitagliptin, and linagliptin.
b) Studies had the human subjects investigated.
c) Studies had to be either randomized/quasi-randomized controlled trials, controlled clinical trials, prospective observational trials with control groups, or retrospective trials.
d) Studies had to be reported in peer-reviewed academic journals or scientific conferences.
e) Language used in articles had to be English.

Two researchers separately completed the study screen. When disagreements arose, a discussion was carried out to arbitrate by another independent reviewer. The below data were obtained, including author information, descriptive data, site country, research design, medication, dosage intensity, duration of diabetes, HbA1c levels, and overall adverse event outcomes. Furthermore, contact was attempted in the studies without quantitative data to access the study’s corresponding authors.

### Quality assessment

The risk of bias was assessed for enrolled trials through Cochrane’s risk of bias assessment tool for randomized controlled trials (13). Methodology quality was evaluated by two reviewers separately. When disagreements arose, an arbitration was carried out by another independent researcher.

### Statistical analysis

Normally distributed data are expressed as the mean ± standard deviation (M ± S.D). Upon a random-effects model (13), Comprehensive Meta-analysis version 2.0 was applied for analyses. Briefly, the pooled prevalence rate was estimated and heterogeneity was imputed by calculating I^2^ statistics. The heterogeneity with a 0%–25% threshold of I^2^ statistics was identified as negligible, 25%–75% moderate, and ≥75% substantial. Then data distribution and global prevalence of HbA1c levels and adverse events between the groups receiving evogliptin, sitagliptin, and linagliptin were conducted. We presented the prevalence, 95% confidence interval (CI), significance level, and heterogeneity. Besides, publication bias analysis was performed by evaluating Duval and Tweedy’s trim and fill method (14). This method presents a nuanced perspective regarding the overall effect, and whether it would shift in case the apparent bias was removed. The analysis is characterized by the imputation of studies from either side of the plotted graph to identify any unbiased effect. Differences below 5% were defined as significant.

## Results

**Figure 1:**
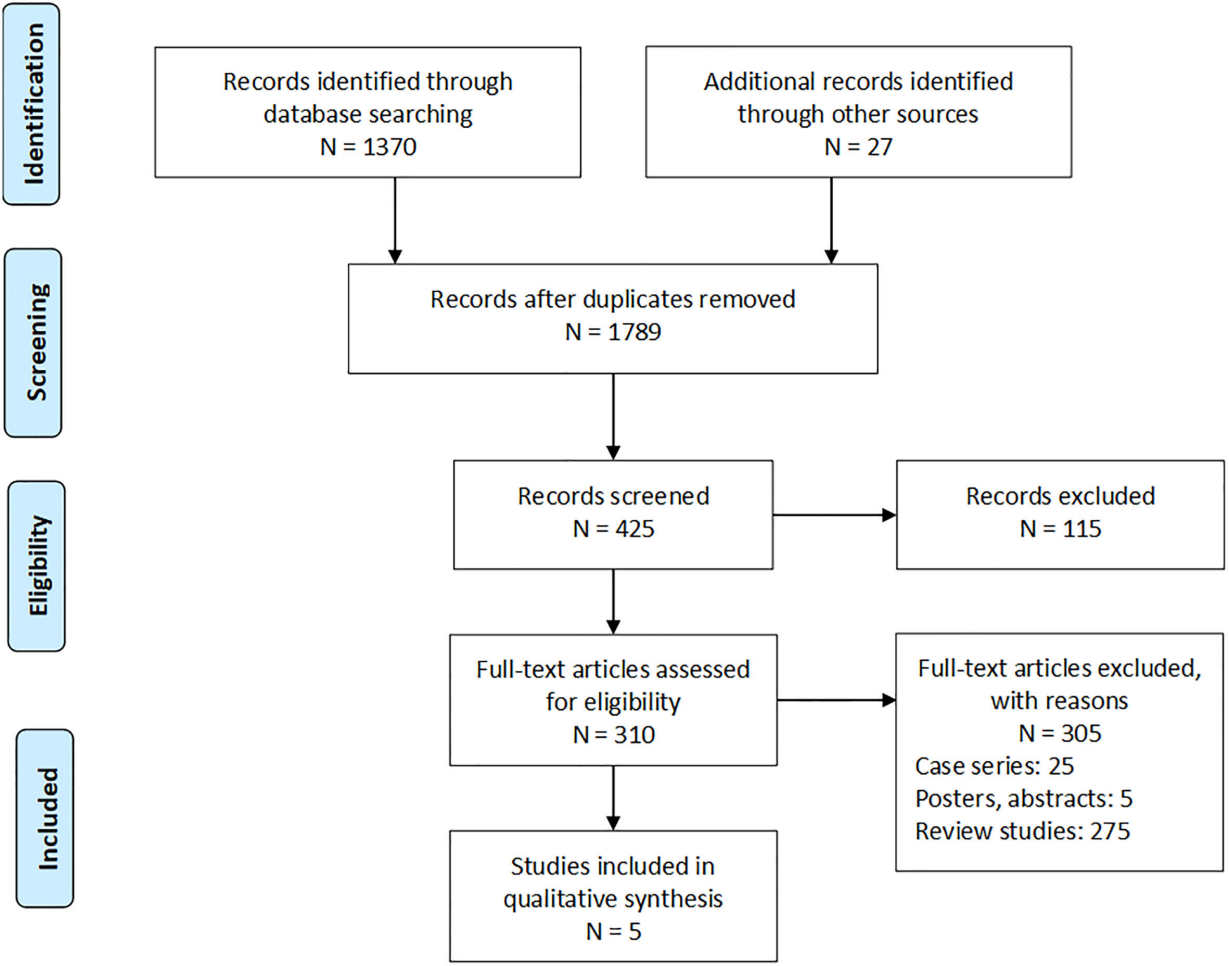
PRISMA flowchart.

A systematic search of four databases yielded 1,370 studies, and a further 27 studies were filtered from the article bibliographies (**Figure 1**). After screening, five studies remained that matched the inclusion criteria, which all were randomized controlled studies, and the analyzed data at a detailed level were displayed in **Table 1**.

**Table 1:** Details of the included studies.

Study	Country	Study design	Period	Group and drug strength (once daily)	N of subjects (female/male)	Age (M ± S.D years)	Duration of diabetes (M ± S.D years)	Hb1Ac levels (M ± S.D %)	Follow-up	Adverse events (n)
Kim et al. (8)	Korea	RCT	12 weeks	Evogliptin, 5 mg	100 (39/61)	56.6 ± 10.7	4.1 ± 4.4	7.5 ± 0.7	12 weeks	30
				Linagliptin, 5 mg	104 (46/54)	55.6 ± 10.2	3.7 ± 4.1	7.6 ± 0.7	12 weeks	42
Cercato et al. (10)	Brazil	RCT	12 weeks	Evogliptin, 5 mg	36 (19/16)	53.1 ± 11.5	1.1 ± 2.8	8.8 ± 0.8	Non	24
				Sitagliptin, 100 mg	39 (18/11)	52.1 ± 10.4	1.1 ± 2.2	8.9 ± 0.9	Non	20
Ajmani et al. (9)	India	RCT	24 weeks	Evogliptin, 5mg	92 (43/49)	49.3 ± 7.5	2.5 ± 3.1	8.2 ± 0.8	Non	28
				Sitagliptin, 100 mg	92 (44/48)	51.4 ± 8.7	2.9 ± 3.6	8.2 ± 0.7	Non	33
Hong et al. (11)	Korea	RCT	24 weeks	Evogliptin, 5 mg	112 (61/51)	57.6 ± 9.4	8.5 ± 5.5	7.4 ± 0.7	52 weeks	50
				Sitagliptin, 100 mg	110 (58/52)	57.3 ± 9.3	7.9 ± 4.9	7.4 ± 0.7	52 weeks	43
Park et al. (15)	Korea	RCT	24 weeks	Evogliptin, 5 mg	80 (34/46)	56.8 ± 9.8	4.2 ± 4.1	7.2 ± 0.6	Non	28
				Control group	80 (41/39)	57.6 ± 11	4.7 ± 3.8	7.2 ± 0.5	Non	26

RCT, randomized controlled trial; M, mean; S.D, standard deviation.

### Participant information

In all, 830 (female/male: 403/427) participants with T2DM were included in the five studies. A total of 419 (female/male: 196/223) patients received evogliptin, whereas 231 (female/male: 120/111) patients received sitagliptin. Linagliptin was given in one study, including a total of 100 (female/male: 46/54) patients (8), and one study included a control group with 80 (female/male: 41/39) patients (15). The mean age of all subjects was 54.7 ± 3.0 years. Here, the age of the group getting evogliptin was 54.6 ± 3.4 years, and the comparator group was 54.8 ± 2.8 years.

### Quality assessment for enrolled trials

**Figure 2:**
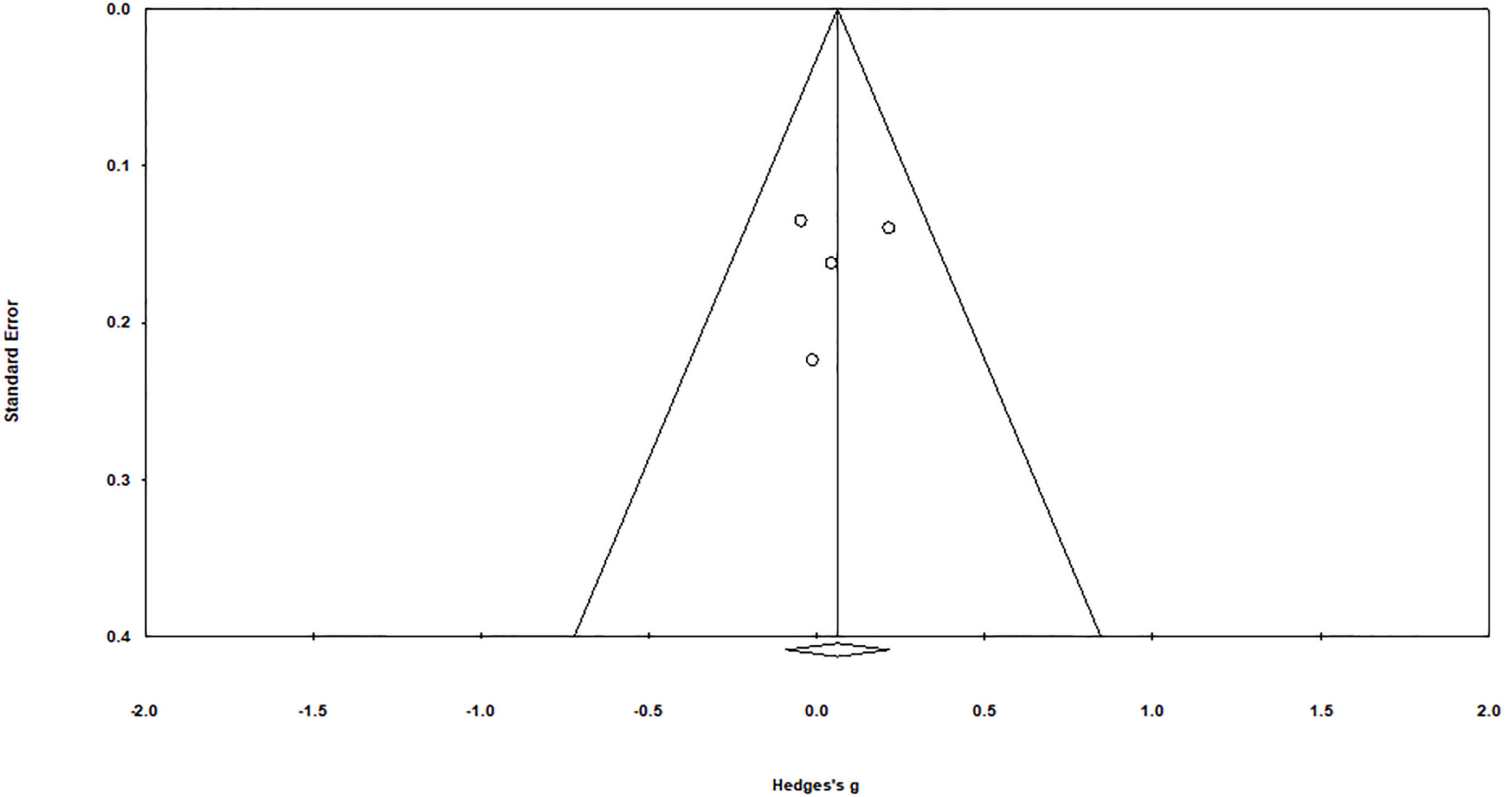
Assessing the publication bias through Duval & Tweedy’s trim and fill method.

**Figure 3:**
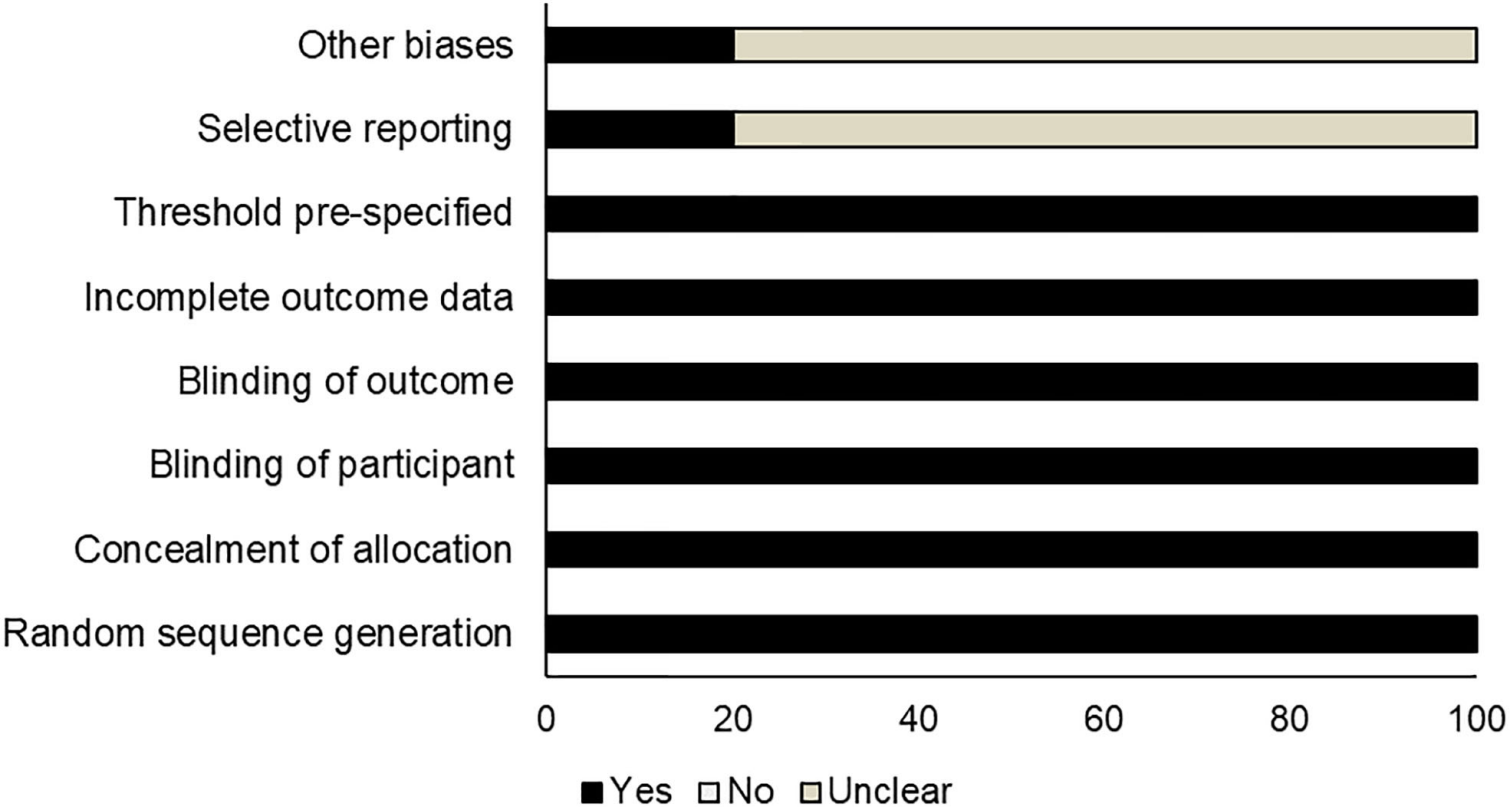
Risk assessment of bias.

Risk assessment of methodological bias was evaluated, with results presented in **Table 2**. We discovered a low overall risk of the pooled studies (**Figure 2**), and selective reporting and other biases were considered the primary biasing sources (**Figure 3**).

**Table 2:** Risk assessment of bias within studies.

	Random sequence generation	Concealment of allocation	Blinding of participant	Blinding of outcome	Incomplete outcome data	Threshold pre-specified	Selective reporting	Other biases
Kim et al. (8)	+	+	+	+	+	+	?	?
Cercato et al. (10)	+	+	+	+	+	+	?	?
Ajmani et al. (9)	+	+	+	+	+	+	?	?
Hong et al. (11)	+	+	+	+	+	+	?	?
Park et al. (15)	+	+	+	+	+	+	+	+

The signs “+” and “?” represented the low risk and unclear risk of bias, respectively.

### Publication bias

Duval and Tweedy’s trim and fill method were employed to determine missed studies complying with the random effect model on both sides of the funnel plot and observed two studies lacking of the right side of the mean effect (**Figure 3**). The overall random effect models determined the point estimate was 0.06 (95% CI: -0.09 to 0.21) in all pooled studies. In contrast, the trim and fill method imputed point estimates were 0.14 (95% CI: 0.008 to 0.27).

### Meta-analysis report

#### HbA1c level and adverse effects

**Figure 4:**
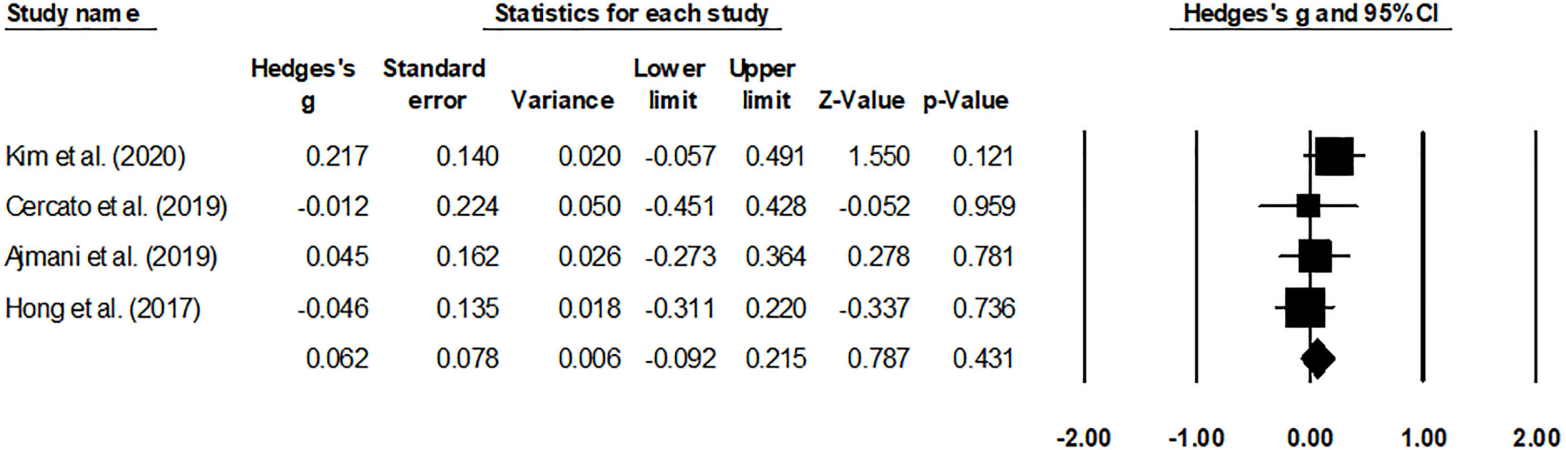
Forest plot for the HbA1c levels. Negative effect sizes denote higher HbA1C levels for the drug sitagliptin/linagliptin and positive effect sizes for evogliptin.

**Figure 5:**
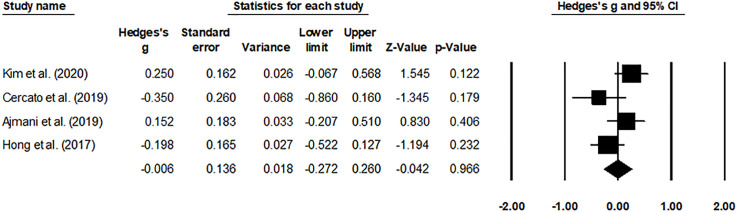
Forest plot for the adverse events. Negative effect sizes denote higher adverse event levels for the drug sitagliptin/linagliptin and positive effect sizes for evogliptin.

The HbA1c levels (**Figure 4**) and adverse effects (**Figure 5**) of the pooled studies were evaluated. Here, one study had compared the effects of evogliptin with linagliptin (8), whereas the other three studies had compared the efficiency of evogliptin to sitagliptin (9–11). Regarding the HbA1c level reduction, evogliptin was non-inferior to sitagliptin/linagliptin with the mean difference of 0.062 (95% CI: -0.092 to 0.215. I^2^: 0%. *P* = 0.431). Regarding the adverse effects, the risk ratio was -0.006 (95% CI: -0.272 to 0.260. I^2^: 1.7%. *P* = 0.966), indicating no significant difference between evogliptin and linagliptin or sitagliptin in affecting the HbA1c level and adverse effects.

## Discussion

Our work demonstrated the comparative efficacy of evogliptin with other DPP-4i drugs such as sitagliptin and linagliptin. We observed a negligible overall difference between the comparative efficacy of evogliptin to sitagliptin and linagliptin in managing HbA1c levels and drug-related adverse events in subjects with T2DM.

Because of the atypical symptoms and progressive nature of T2DM, its management is exceptionally challenging for diabetologists worldwide. Being a heterogeneous metabolic disorder, T2DM develops due to the onset of two significant defects in insulin production and the development of resistance toward insulin (16). The impairment eventually influences how the insulin interacts with the β-cells and results in widespread dysfunction. Scheen explained that one of the primary mechanisms that instigate β-cell deficiency is causing dysfunction within the insulin signaling pathways in the pancreas (4). Additionally, the onset of β-cell insufficiency could have also occurred due to inadequate *in-utero* nutrition as T2DM is highly associated with familial history (for more details see “Thrifty-phenotype hypothesis”) (17). Likewise, the increased presence of blood glucose levels due to impairments in hepatic metabolization and a rise in abdominal adiposity (18) have also been suggested as another mechanism facilitating the onset of T2DM. Together, the metabolic changes related to T2DM and associated co-morbidities eventually worsen the prognostic outcome regarding morbidity and mortality related.

For this metabolic disorder, many factors have recently intensified DPP-4i class drugs such as evogliptin (8, 11). Firstly, evogliptin promoted selective dipeptidyl peptidase-4 inhibition at a high rate of >80%, even after 36-h of a small dosage (19). Secondly, the improved pharmacokinetic profile of evogliptin allows daily dosing with a high safety profile (reduced adverse events) (10). Thirdly, the administration of drugs is also favored because of its capability to avoid renal metabolism, making it safe for patients suffering from chronic nephropathy. Fourthly, evogliptin has also been widely recommended based on its superior capabilities to reduce blood glucose levels compared to other DPP-4i class drugs (8). For example, Ajmani et al. reported that a 5-mg dosage of evogliptin mediated the HbA1c levels far more strongly than a 100-mg dosage of sitagliptin at 12-week and 24 weeks of administration (9). The authors also mentioned that evogliptin also resulted in a body weight reduction of -0.29 ± 2.2 kg compared to -0.33 ± 2.1 kg for the group receiving sitagliptin at 24 weeks of administration. Similarly, Kim et al. reported the beneficial comparative influence of evogliptin compared to linagliptin in a multicenter randomized controlled trial study. The authors mentioned that evogliptin reduced the levels of N-acetyl β D-glucosaminidase after both 12-week and 24-week treatment (8). Evogliptin reduced HbA1c levels by 0.85 percent after 12 weeks and 0.94 percent after 24 weeks by increasing glycemic index variability. Cercato et al. confirmed that 5-mg evogliptin could achieve the HbA1c lowering effect of 100-mg sitagliptin (10). In comparison, Hong and others demonstrated the non-inferiority of evogliptin to sitagliptin in reducing the level of HbA1c (11). Therefore, when diabetologists face the choice of DPP-4i for long-term glycemic control, the priority of this class of drugs cannot be determined. In this review, several included trials were analyzed. They showed that evogliptin was non-inferior to sitagliptin/linagliptin with a mean difference of 0.062 (95% CI: -0.092 to 0.215. I^2^: 0%. *P* = 0.431) regarding the HbA1c level reduction, indicating no significant difference between evogliptin and linagliptin or sitagliptin in affecting the HbA1c level. These results suggested that for diabetologists, evogliptin, and linagliptin or sitagliptin have the same recommendation for reducing HbA1c in T2DM patients.

Besides, an attempt was made to reach a consensus on the drug-related adverse events between evogliptin and sitagliptin/linagliptin. Here, inconclusive evidence regarding the overall adverse event outcome was reported in this review. Cercato and Hong reported that a 100-mg dosage of sitagliptin had a lower adverse event outcome than 5 mg of evogliptin (10, 11). In contrast, Ajmani and Kim demonstrated a reduced adverse event outcome for the evogliptin group and no difference between the evogliptin group as compared to other DPP-4i class drugs, respectively (8, 9). Our meta-analysis backed up the latter findings by reporting an insignificant risk ratio of -0.006 (95% CI: -0.272 to 0.260. I^2^: 1.7%. *P* = 0.966) between evogliptin and sitagliptin/linagliptin, indicating no significant difference in affecting the adverse effects. These results indicated that for diabetologists, evogliptin and linagliptin or sitagliptin have the same recommendation for affecting the adverse effects of T2DM patients.

However, there were a few limitations that persisted in our study. Firstly, we failed to register the study in any review repository such as PROSPERO because of the extended registration time of over 1 year and the COVID-19 pandemic, which may cause doubts concerning the validity of this study. Secondly, the scarcity of data with pooled studies might bias the previous interpretation of HbA1c and adverse event outcomes. Here, while three studies had reported the comparative efficacy of evogliptin with other drugs in the DPP-4i class after 12 weeks, one study reported the comparative accuracy after 24 weeks. Despite the lack of observed heterogeneity in the study, a cautious interpretation of these results is highly suggested for readers, and more multicenter randomized controlled studies are needed in the future in open access data repositories. Evaluating these outcomes will help diabetologists establish optimal practical strategies for managing T2DM with DPP-4i class drugs.

## Conclusion

Based on limited evidence, this study presents preliminary proof of HbA1c reduction and drug-related adverse events between evogliptin and sitagliptin/linagliptin, and demonstrated that evogliptin was non-inferior to sitagliptin/linagliptin about HbA1c reduction and drug-related adverse events for T2DM patients. The relevant results may contribute to establishing optimum practice guidelines for managing T2DM and its associated complications using DPP-4i class drugs.

## Data availability statement

The original contributions presented in the study are included in the article/supplementary material. Further inquiries can be directed to the corresponding author.

## Author contributions

QT conceived and designed and wrote the paper. WP and LP consulted the literature and analyzed the data. All authors contributed to the article and approved the submitted version.

## Conflict of interest

The authors declare that the research was conducted in the absence of any commercial or financial relationships that could be construed as a potential conflict of interest.

## Publisher’s note

All claims expressed in this article are solely those of the authors and do not necessarily represent those of their affiliated organizations, or those of the publisher, the editors and the reviewers. Any product that may be evaluated in this article, or claim that may be made by its manufacturer, is not guaranteed or endorsed by the publisher.
